# Cysteine-mediated structural stabilization of the tetrameric GlpF

**DOI:** 10.1007/s00249-025-01803-6

**Published:** 2025-11-03

**Authors:** Christine Siligan, Sascha Gratzl, Kristyna Pluhackova, Nikolaus Goessweiner-Mohr, Peter Pohl, Andreas Horner

**Affiliations:** 1 Institute of Biophysics, Johannes Kepler University Linz, Linz, Austria; 2 Stuttgart Center for Simulation Science, Cluster of Excellence EXC 2075, University of Stuttgart, Stuttgart, Germany

**Keywords:** Aqua(glycerol)porin, *Escherichia coli*, GlpF, Protein stability, Cysteine mutations, MD simulations, NanoDSF

## Abstract

The stability of membrane proteins depends on amino acid-specific interactions within the protein structure, its surroundings, e.g. the lipid membrane and ionic solutions, and their substrates. A molecular understanding of the factors affecting protein stability is essential for comprehending the respective structure–function relationships. This study aims to investigate the influence of native cysteine residues, ions, and glycerol on the stability of GlpF, the aqua(glycerol)porin of *E. coli*, under well-controlled conditions. To this end, the wild-type protein and its variant with four cysteine residues in the transmembrane helix bundle mutated to glycines were overexpressed and purified. The thermal unfolding of GlpF occurs at a transition temperature of 59.2 ± 1.4 °C, corresponding to an apparent Gibbs free energy of unfolding of approximately 10 kcal/mol. In contrast to the wild-type protein, the mutated GlpF exhibits a second unfolding transition, lowered by approximately 10 °C, and a decreased Gibbs free energy of unfolding of 5 kcal/mol. Our findings highlight that divalent ions and glycerol have a more pronounced stabilizing effect on the mutated GlpF than the wild-type protein. The structural consequences of cysteine to glycine mutations in terms of transmembrane helix rearrangement, the inter- and intra-chain H-bond network, and the reduced inter-chain interaction free energy, as well as intra-chain electrostatic energy and van der Waals energy, are revealed by molecular dynamics simulations. In conclusion, our findings illustrate the significance of cysteine residues distant from each other on protein oligomerization and stability, and the amplified impact of (de)stabilizing agents on less stable protein variants.

**Supplementary Information:**

The online version contains supplementary material available at https://doi.org/10.1007/s00249-025-01803-6.

## Introduction

Membrane proteins are essential in the selective transport of solutes across cellular membranes. In contrast to the growing molecular comprehension of their structure and function (Levental and Lyman 2023; Eriksson et al. 2013; Wachlmayr et al. 2023; Horner and Pohl 2018), there is insufficient knowledge concerning the principles governing the oligomeric assembly of membrane proteins and the functional consequences of those dynamic processes. As illustrated by aquaporins, one of the most extensively studied transmembrane protein families facilitating the selective permeation of water (Saparov et al. 2005; Horner et al. 2015) and small neutral solutes (Fu et al. 2000; King et al. 2004), the tetrameric structure is stabilized through hydrophobic interactions, hydrogen bonds (H-bonds), and salt bridges at the protein–protein interface (Goessweiner-Mohr et al. 2022). The latter are situated predominantly at the interface between the lipid bilayer and the aqueous phase (Goessweiner-Mohr et al. 2022). Furthermore, the tertiary structure is reinforced by a substantial internal H-bond network, which defines and stabilizes the structure and dynamics of the pores, including the aromatic and Arg-containing (Ar/R) selectivity filter and the highly conserved NPA regions (Goessweiner-Mohr et al. 2022). Both of these are crucial for the selective transport of solutes and the rejection of protons, despite the presence of a continuous file of water molecules within the pore (Eriksson et al. 2013; Beitz et al. 2006; Li et al. 2011; Groot and Grubmuller 2005). Furthermore, the stability and functionality of membrane proteins are dependent on the interactions between lipids and proteins (Laganowsky et al. 2014). The binding of lipids at weakly stabilized interfaces has been demonstrated to stabilize loosely interacting protomers (Gupta et al. 2017) and changes in the lipid composition can alter the overall oligomeric state of membrane proteins (Anderluh et al. 2017). Furthermore, the interaction of negatively charged lipids with AQPs and the binding of cardiolipin in the crevice at the contact site between monomers has been demonstrated to affect AQP stability (Pluhackova and Horner 2021) and function (Laganowsky et al. 2014).

In general, oligomerization may result in structural and/or proteolytic stability, functional diversity, regulatory mechanisms, and the formation of binding cavities (Neumann et al. 2014). For example, the two AQP family members of *Escherichia coli*, AqpZ, and GlpF reduce their activity upon monomerization (Schmidt and Sturgis 2017; Cymer and Schneider 2012; Klein et al. 2019; Trefz et al. 2018). The model protein GlpF is an aqua(glycero)porin that, in addition to its exceptional water permeability (Horner et al. 2015), facilitates the permeability of small uncharged solutes, including glycerol and urea (Fu et al. 2002). Compared to other AQPs, the residues that line the channel are highly mobile, frequently impeding the water flow through the pore of GlpF (Goessweiner-Mohr et al. 2022; Pluhackova et al. 2022). In addition to exhibiting a diminished ribitol flux, monomeric GlpF was reported to reduce resistance to proteolysis in *E. coli* (Cymer and Schneider 2010) and diminished stability relative to the tetrameric protein (Galka et al. 2008). In the case of human *h*AQP2 and *h*AQP4, where the monomeric variant exhibited either a loss of function (Marr et al. 2001) or similar water permeability (Kitchen et al. 2016), respectively, tetramer destabilization resulted in the inability of the proteins to relocate to the plasma membrane (Kitchen et al. 2016; Kamsteeg et al. 1999).

Several methods and techniques are available to study aggregation, (un)folding, and stability of membrane proteins. Besides the possibility to asses membrane protein stability by mass spectrometry (Gupta et al. 2017) or single-molecule fluorescence microscopy (Anderluh et al. 2017; Chadda et al. 2016), the majority of the standard methods are based on protein unfolding in response to a thermal or chemical gradient (Galka et al. 2008; Swamy-Mruthinti et al. 2013; Baturin et al. 2014; Plasencia et al. 2011; Hagstromer et al. 2023; Oberg et al. 2011; Roche et al. 2017; Kozono et al. 2003). To assess the thermostability of AQPs, a range of techniques were employed, including SDS-PAGE, with temperature-exposed protein samples (Galka et al. 2008; Swamy-Mruthinti et al. 2013; Baturin et al. 2014), semi-native and native PAGE (Ishiguro and Fujisawa 2023), Trp fluorescence (Plasencia et al. 2011), scattering/absorbance (Swamy-Mruthinti et al. 2013), differential scanning fluorimetry (DSF) (Hagstromer et al. 2023), far-UV CD-spectroscopy (Galka et al. 2008; Swamy-Mruthinti et al. 2013; Plasencia et al. 2011; Hagstromer et al. 2023; Oberg et al. 2011; Roche et al. 2017), and stopped-flow methodology (Kozono et al. 2003). It is noteworthy that these methods examine slightly different effects during thermally induced protein unfolding. Scattering/absorbance measurements (Swamy-Mruthinti et al. 2013) and SDS-PAGE, semi-native and native PAGE track protein oligomerization and unspecific aggregation (Galka et al. 2008; Swamy-Mruthinti et al. 2013; Baturin et al. 2014; Failer et al. 2003), whereas Trp fluorescence (Plasencia et al. 2011) and DSF (Hagstromer et al. 2023) examine the folding of the quaternary, tertiary, and possibly secondary structure. Far-UV CD spectroscopy (Galka et al. 2008; Swamy-Mruthinti et al. 2013; Plasencia et al. 2011; Hagstromer et al. 2023; Oberg et al. 2011; Roche et al. 2017) assesses the thermal stability of the secondary structure, and stopped-flow methodology monitors the thermally induced loss of protein function (Kozono et al. 2003). It is reasonable to assume that there is a cooperative thermostability effect in AQPs; however, the melting temperatures of the aforementioned features need not necessarily concur as illustrated for GlpF (Galka et al. 2008). It was demonstrated that the secondary structural elements exhibited a markedly reduced melting temperature in comparison to the tertiary and quaternary structural elements (Galka et al. 2008). Thus far, AQPs have been observed to undergo irreversible unfolding as a consequence of thermal stress (Swamy-Mruthinti et al. 2013; Plasencia et al. 2011). The thermal stability of the spinach aquaporin *So*PIP2;1 was shown to depend on its incorporation into lipid bilayers (Plasencia et al. 2011). Similarly, protein glycosylation was demonstrated to affect the thermal stability of hAQP10 (Oberg et al. 2011) and hAQP2 (Roche et al. 2017). Additionally, protein mutations and pH, as exemplified by hAQP2 (Hagstromer et al. 2023) and GlpF (Galka et al. 2008), respectively, were proven to influence the thermal stability of AQPs. The addition of trimethylamine N-oxide (TMAO) (Baturin et al. 2014) and the choice of detergent (Galka et al. 2008) were also shown to affect the thermal stability of GlpF.

In this study, we compare the melting temperatures of the GlpF wild-type protein with a destabilized variant, shown to facilitate passive water permeability (Horner et al. 2015), and calculate the apparent Gibbs free energy of unfolding of these proteins utilizing nanoDSF. Furthermore, the impact of diverse environmental variables, including the presence of divalent ions, the substrate glycerol, elevated concentrations of sodium chloride, and a range of detergents on the thermal stability of both variants is investigated. This provides unique insight into the importance of diverse factors on GlpF and a mutationally destabilized variant. In addition, we use all-atomistic molecular dynamics simulations to gain molecular insight into the structural and energetic variations between the two variants.

## Material and methods

### GlpF overexpression and purification

*E. coli* C43 cells were transfected with pTrcHis-GlpF (wt, mutG—C11^1.−9^/28^1.8^/80^3.−16^/99^3.3^G, mutA–C11^1.−9^/28^1.8^/80^3.−16^/99^3.3^A) and grown in LB containing 100 µg/L Ampicillin at 37 °C till O.D. 600 nm ~ 0,8. GlpF production followed a previously published protocol (Saparov et al. 2005) with minor modifications. Expression of GlpF constructs was induced with 1 mM IPTG for 3 h followed by centrifugation for 10 min at 7000 g. Cell pellets were lysed in 50 ml Lysis Buffer (100 mM KPh Buffer pH 8.0, 1 mM MgSO4) applying 3 cycles of 15000 psi with an Emulsi Flex homogenizer at 4 °C. After clearing the lysate by centrifugation for 10 min at 7000 g, the supernatant was separated into membrane pellet and soluble fraction by ultracentrifugation for 100 min at 135,000 g at 4 °C. Membrane pellets were solubilized in solubilization buffer (100 mM KPh buffer pH 8.0, 200 mM NaCl, 10% glycerol, 20 mM imidazole, 5 mM β-mercaptoethanol, 3% octylglycopyranoside (OG)) for 2 h at 4 °C, followed by ultracentrifugation for 100 min at 135,000 g at 4 °C to pellet the lipid fraction. The supernatant containing solubilized membrane proteins were affinity purified using Ni–NTA resin. After incubation of membrane proteins with Ni–NTA resin for 16 h at 4 °C, columns were washed with 100 times BV using wash buffer (100 mM KPh pH 7.0, 300 mM NaCl, 10% glycerol, 20 mM imidazole, 2% OG). GlpF was eluted from the columns in elution buffer (100 mM KPh pH 7.0, 200 mM NaCl, 10% glycerol, 1 M imidazole, 2% OG). Protein concentration was measured using Bradford assays (Kruger 2002) and purity of GlpF was verified by SDS-PAGE. Since membrane proteins tend to aggregate when completely denatured due to their hydrophobic nature, samples were mixed with SDS-loading buffer immediately before loading the gels without incubation at high temperatures. Thus, the proteins run faster through the gels than their calculated molecular weights suggest (Rath and Deber 2013). Double bands can be interpreted as folded and partially unfolded (secondary structure) proteins. The samples were separated on an Äkta pure chromatography system (Cytiva) using a Superdex 200 increase 10/300 column (Cytiva) in running buffer (100 mM KPh pH 7.0, 300 mM NaCl, 2% OG). The flow rate was set to 0.4 ml/min with 0.35 ml fractions collected.

### Nano differential scanning fluorometry (nanoDSF)

Tryptophan fluorescence measurements (absorption at 330 nm and 350 nm) were performed on Prometheus NT.48 (NanoTemper GmbH, Munich, Germany) using standard-grade glass capillaries filled with approximately 15 µl sample volume. Prior to capillary loading, different sample conditions were prepared. After the addition of additives or adjusting the concentration, samples were centrifuged for 15 min at 14,000 g at 4 °C to remove aggregates. The excitation light was adjusted to record fluorescence readings at appropriate intensity (arbitrary unit). Samples were submitted to a temperature ramp ranging from 15 to 95 °C with a slope of 1 °C/min. Data were fitted using the online version of MoltenProt (https://spc.embl-hamburg.de/app/moltenprot) (Burastero et al. 2021; Kotov et al. 2021) with the equilibrium two-state and the equilibrium three-state model for comparison. As these models are only applicable for reversible unfolding events not compatible with large molecular weight membrane proteins like AQPs, fitted parameters are only apparent and not absolute thermodynamic parameters (Kotov et al. 2019).

The equilibrium two-state model (ES2) assumes that the protein exists in either the native (N) or unfolded (U) state and that there is an equilibrium between the folding and unfolding reactions (N ⇆ U) (Bedouelle 2016; Santoro and Bolen 1988).

$$F\left(T\right)=\frac{{k}_{n}T+{b}_{n}+({k}_{u}T+{b}_{u})\bullet \mathrm{exp}\left(\frac{\Delta {H}_{m}}{R}(\frac{1}{{T}_{m}}-\frac{1}{T})\right)}{1+\mathrm{exp}\left(\frac{\Delta {H}_{m}}{R}(\frac{1}{{T}_{m}}-\frac{1}{T})\right)}$$

where ${k}_{n}$, ${b}_{n}$ are the slope and intercept of the pre-transition (native) baseline, respectively, ${k}_{u}$, ${b}_{u}$ are the slope and intercept of post-transition (unfolded) baseline, respectively, R is the universal gas constant, and $\Delta {H}_{m}$ is enthalpy of unfolding at melting temperature ${T}_{m}$. ΔG of unfolding is estimated using

$$\Delta {G}_{T}\left(\Delta H,{T, T}_{m}\right)=\Delta H\left(1-\frac{T}{{T}_{m}}\right)-{T}_{m}+T\bullet \mathrm{log}\left(\frac{T}{{T}_{m}}\right)$$

The equilibrium three-state model (ES3) assumes that the protein exists in three possible states: native (N), unfolded intermediate (I), and unfolded (U) state (Mazurenko et al. 2017).

$$F\left(T\right)=\frac{{k}_{n}T+{b}_{n}+{k}_{I}\bullet \mathrm{exp}\left(\frac{\Delta {H}_{m1}}{R}(\frac{1}{{T}_{1}}-\frac{1}{T})\right)+({k}_{u}T+{b}_{u})\bullet \mathrm{exp}\left(\frac{\Delta {H}_{m1}}{R}(\frac{1}{{T}_{1}}-\frac{1}{T})\right)\bullet \mathrm{exp}\left(\frac{\Delta {H}_{m2}}{R}(\frac{1}{{T}_{2}}-\frac{1}{T})\right)}{1+\mathrm{exp}\left(\frac{\Delta {H}_{m}}{R}(\frac{1}{{T}_{m}}-\frac{1}{T})\right)}$$

where ${k}_{n}$, ${b}_{n}$ are the slope and intercept of the pre-transition (native) baseline, respectively, ${k}_{u}$, ${b}_{u}$ are the slope and intercept of post-transition (unfolded) baseline, respectively, ${k}_{I}$ is the signal slope for the (short-lived) I state, R is the universal gas constant, $\Delta {H}_{m1}$ and $\Delta {H}_{m2}$ are the enthalpy of unfolding at melting temperatures ${T}_{1} (N\leftrightarrows I)$ and ${T}_{2} (I\leftrightarrows U)$, respectively.

### Molecular dynamics simulations

The all-atom molecular dynamics (MD) simulations of the tetrameric wild-type GlpF we have previously published (Pluhackova et al. 2022) were analyzed and compared with the here performed all-atom MD simulations of the mutant C11^1.−9^/28^1.8^/80^3.−16^/99^3.3^G (mutG), also in the tetrameric form. Each GlpF tetramer was surrounded by a 3-component model of *E. coli* polar lipid extract consisting of 1-palmitoyl-2-oleoyl-sn-glycero-3-phosphoethanolamine (POPE), 1-palmitoyl-2-*cis*−9,10-methylene-hexadecanoic-acid-*sn*-glycero-3-phosphoglycerol (PQPG) (Pandit and Klauda 2012) and 1-[1-*cis*−9,10-methylene-hexadecanoic-acid-2-pamitoyl-*sn*-glycero-3-phospho]−2-[1,2-dipalmitoyl-sn-glycero-3-phospho] -*sn*-glycerol (cardiolipin) in 67:23:10 molar ratio. The protein-membrane systems were solvated by TIP3P water (Jorgensen et al. 1983) and the total charge was neutralized by Na^+^. Using the wt GlpF system prior equilibration, the four cysteines in each chain were mutated in PyMOL (Schrödinger 2020). The simulation workflow followed that of the wt GlpF (Pluhackova et al. 2022). In detail, the CHARMM36(m) force field (Pandit and Klauda 2012; Huang et al. 2017; Klauda et al. 2010) was used and the whole system underwent a three-step energy minimization (freezing the proteins without the mutated residues, the protein backbone, and nothing) of 1000 steps each. The energy-minimized system was then equilibrated by two position restraint simulations. In the first one lasting 20 ns, all but hydrogen atoms of the proteins were position restrained to their original position using a force of 1000 kJ/mol/nm. In the second position restraint simulation of 10 ns length, the backbone atoms of the proteins were restricted with the same force. Thereby, the PME was used to calculate the electrostatics behind 1.2 nm, the van-der-Waals interactions were force-switched to zero between 0.8 and 1.2 nm, the Verlet cut-off scheme (Páll and Hess 2013) with buffer tolerance of 0.005 kJ/mol/ps per atom was used and the bonds to hydrogens were restraint using LINCS (Hess 2008). The Berendsen barostat (Berendsen et al. 1984) controlled the pressure in a semi-isotropic manner to be 1 bar with a time constant of 5 ps and compressibility of 4.5 × 10^–5^ bar^−1^. The Nosé-Hoover thermostat (Evans and Holian 1985) with a time constant of 0.5 ps kept the temperature of the protein/membrane and the solvent separately at 37 °C. The center of mass scaling was applied to the position restraint coordinates and the time step of 2 fs was used. The production run simulation was run in 2 replicas of 3 µs each. Thereby the Parrinello-Rahman barostat was used instead of the Berendsen barostat and the center of mass of the system was linearly removed each 500 steps. No center of mass scaling was applied. The trajectories were saved every 10 ps. All simulations were performed in GROMACS2018 (Abraham et al. 2015). The radius of the pore was estimated using HOLE v2 (Smart et al. 1996). For identification of hydrogen bonding of cysteine SH groups, the distances between the SH hydrogen and each oxygen or nitrogen in its surroundings were measured and counted as a hydrogen bond if the distance was shorter than 0.3 nm (Carugo 2023). Distances among Cα atoms of following residues were used to estimate the distances between the helices: central layer (H1 22, H2 49, H3 97, H4 155, H5 187, HH 204, H6 240), cytoplasmic layer (H1 13, H2 56, H3 87, H4 163, H5, 180, HH 73, H6 248) and periplasmic layer (H1 29, H2 41, H3 105, H4 146, H5 196, HH 214, H6 232). The plots were generated in R (R Core Team 2020).

## Results and discussion

**Fig. 1:**
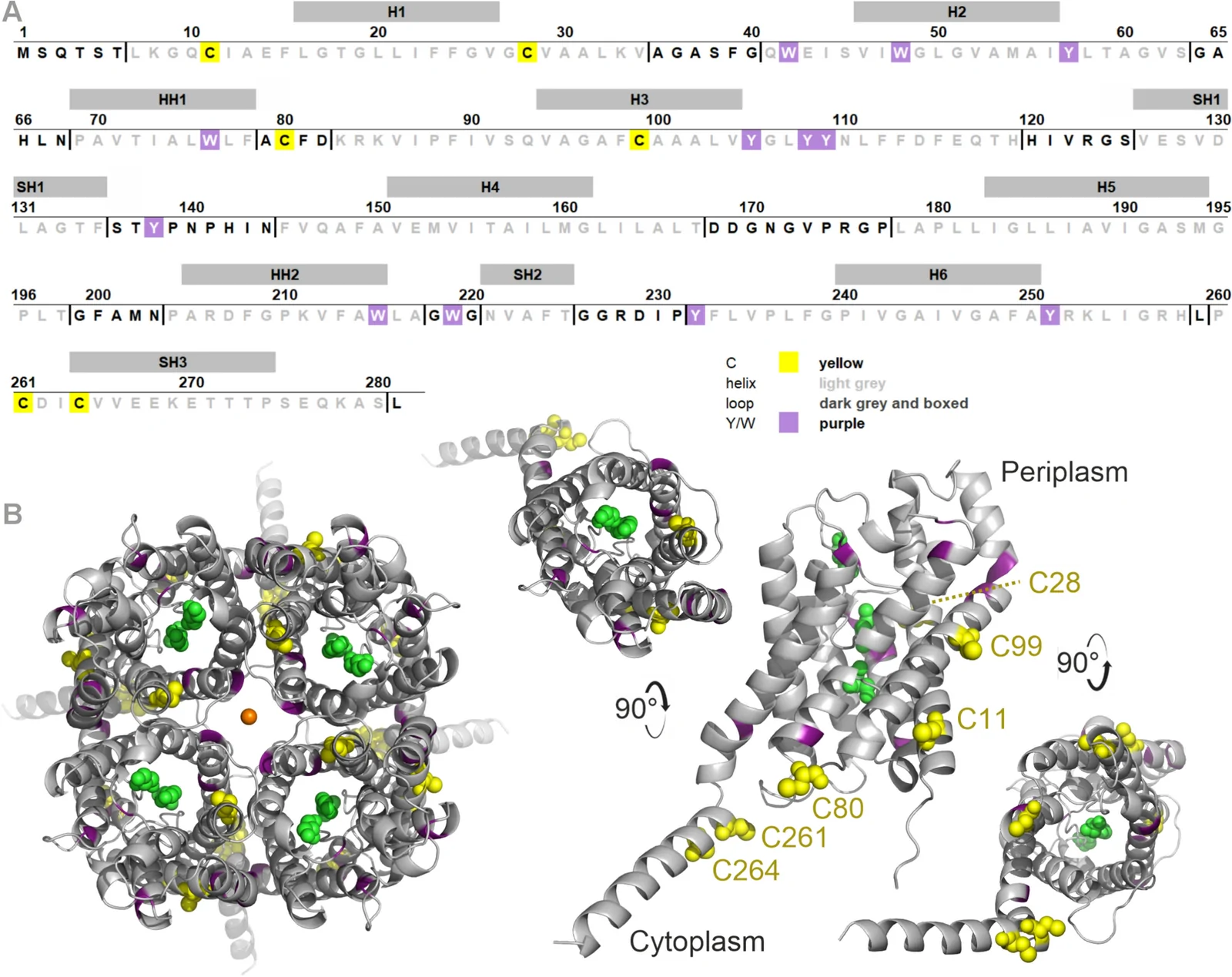
The sequence and structure of GlpF. **A** GlpF (Fu et al. 2000) contains 281 amino acids (29.78 kDa) which fold into six transmembrane helices (H), two half-helices (HH), and according to AlphaFold3 (Abramson et al. 2024) into one C-terminal (cytoplasmic) water-soluble helix (SH3) as well as two short periplasmic water-soluble helices (SH1 & SH2) between H3 and H4, and HH2 and H6, respectively. A comparison between the structural model and the crystal structure (PDB:ID: 1FX8) of GlpF (Fu et al. 2000) is presented in Fig. S1. Helices and loop regions are indicated with grey and black letters, respectively. The six native cysteine residues C11^1.−9^, C28^1.8^, C80^3.−16^, C99^3.3^, C261^6.21^, and C264^6.24^ are highlighted in yellow. The superscript indicators are used to denote the recently introduced general numbering scheme of amino acid positions in AQPs (Goessweiner-Mohr et al. 2022). The intrinsic tryptophan and tyrosine residues important for nanoDSF measurements are indicated in purple. Trp42^2.−9^ is facing the central pore, Trp76^h1.7^ and Trp215^6.−25^ are facing the lipid, and Trp48^2.−3^ is facing the protomer pore. Tyr105^3.9^ and Tyr109^3.13^ are facing the lipid, Tyr57^2.6^ and Tyr108^3.12^ are facing the protomer interface, Tyr138^4.−18^ and Tyr232^6.−8^ are located within a protomer, and Tyr251^6.11^ according to the simulations often makes contacts to the neighboring protomer. The N- and C-termini are oriented towards the cytoplasm. **B** A representation of the GlpF tetramer (AlphaFold3 model), viewed from the periplasm. In the side view, the monomer is oriented with the periplasmic side facing upwards. Additionally, single protomers are depicted from the cytoplasm (bottom right) and the periplasm (middle top). The glycerol molecules and magnesium ion, co-crystallized in the structure with the PDB identifier 1FX8, are superimposed into the model and shown in green and orange spheres, respectively. Cysteine residues are represented by yellow spheres. Tryptophan and Tyrosine residues are highlighted by purple color. Glycerol is bound to the protomer pores, facilitating passive water and small neutral solute permeation. In contrast, Mg^2+^ is bound to the central, sometimes also termed fifth, pore

**Fig. 2:**
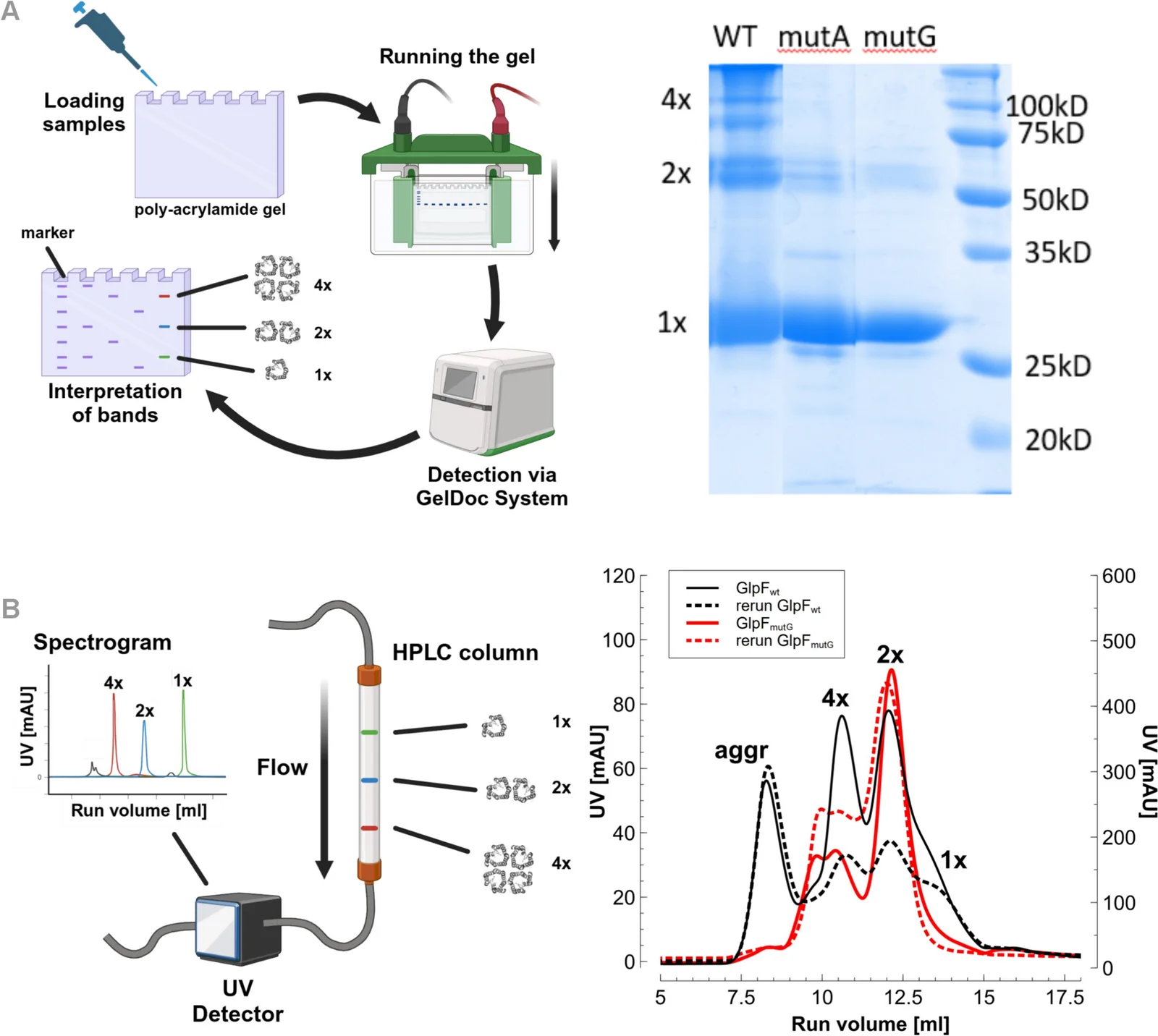
GlpF_muts_ represent destabilized variants of GlpF_wt_. **A** Principles and results of SDS-PAGE. The protein samples are premixed with the loading buffer and subsequently loaded on top of the polyacrylamide gel at room temperature. The application of an electric field to the gel facilitates the separation of the samples according to their respective protein masses. The distance between bands is inversely proportional to the mass of the protein, with smaller proteins migrating the greatest distance. The protein bands are then subjected to Coomassie brilliant blue staining, which is detected via a GelDoc system. The exemplary protein lanes of GlpF_wt_ (wt), GlpF_mutA_ (mutA), and GlpF_mutG_ (mutG) are displayed adjacent to the marker lane on the right. Monomeric bands of considerable intensity at approximately 27 kDa are discernible for all three proteins. In contrast, the presence of strong bands at higher molecular weights is absent for both mutants. **B** Size exclusion chromatography is a technique used to separate molecules based on their size. The flow of larger molecules through the Superdex 200 HPLC column is solely dependent on the flow rate, whereas smaller molecules are retarded through the heteroporous beads. The visualization of proteins is achieved by detecting the aromatic amino acids at a wavelength of 280 nm on a chromatogram. The exemplary chromatograms for GlpF_wt_ (black line) and GlpF_mutG_ (red line) are presented on the right. Run volumes at approximately 8 ml, 11 ml, 12 ml, and 14 ml correspond to aggregates, tetramers, dimers, and monomers, respectively. Dotted lines indicate the chromatograms of the reloaded and up-concentrated tetrameric fractions

The impact of transmembrane cysteine residues (see Fig. 1) on the oligomeric stability of GlpF was evaluated through mutagenesis. The cysteine residues at positions 11, 28, 80, and 99 were mutated either into glycines (GlpF_mutG_) or alanines (GlpF_mutA_). As demonstrated in Fig. 2A GlpF_mutG_ and GlpF_mutA_ manifest a more pronounced monomeric fraction on the SDS-PAGE compared to the wild-type (GlpF_wt_) protein, indicating a destabilized oligomeric assembly. Thereby, the band patterns observed for the alanine and glycine mutants are similar. Conversely, the wild-type protein displays multiple bands of considerable height, indicative of the presence of higher-order oligomers. In our previous research, we functionally characterized GlpF_mutG_ (Horner et al. 2015). As both mutants exhibited similar behavior on SDS-PAGE, we further utilized GlpF_mutG_ as a destabilized GlpF variant throughout the current manuscript. The destabilization of GlpF through the C11/28/80/99G mutations is further illustrated by an anti-His antibody staining of the solubilized proteins (see Fig. S2), where higher oligomeric bands are absent for GlpF_mutG_. Moreover, a chemo stability test with increasing detergent concentrations revealed a destabilization of GlpF_mutG_ at higher OG as well as sodium dodecyl sulfate (SDS) concentrations (see Fig. S3).

As demonstrated in the SDS-PAGE, the chromatogram of size exclusion chromatography (Fig. 2B) of GlpF_wt_ in OG micelles also exhibited the presence of multiple oligomeric assemblies. Despite minor variations in peak position and height between protein purifications, the peaks observed at run volumes between 10 and 15 ml most likely correspond to the tetrameric, dimeric, and monomeric protein assemblies, respectively. However, precise assignment remains challenging due to the run volume-dependent nature of the assay, which is contingent on the exact protein-to-detergent micelle size ratio (Kunji et al. 2008). The peak at approximately 8 ml could be identified as an aggregate peak. In contrast, GlpF_mutG_ chromatograms exhibit a negligible aggregate peak at approximately 8 ml and a less pronounced oligomeric state distribution with diminished tetrameric and monomeric peak fractions. It is noteworthy that reloading the tetrameric peaks yielded similar peak patterns again in the subsequent chromatograms, suggesting a dynamic equilibrium of oligomeric states in the detergent micelles for GlpF_wt_ and GlpF_mutG_.

Subsequently, the thermostability of GlpF_wt_ and the destabilized variant GlpF_mutG_ was evaluated through the utilization of nanoDSF. GlpF contains five tryptophan (Trp) residues per monomer sequence, which exhibit an absorption maximum at 280 nm and emission at around 330 nm within protein structures (Teale 1960). However, the emission spectra of these residues are subject to change depending on their chemical environment. For instance, a shift in polarity (Sun et al. 2010), occurring if Trp is exposed to a lipid/detergent environment, protected by the natural fold, or if the hydrophobic side chains are exposed to the aqueous solution, will result in a different emission spectrum. In the latter case, the fluorescence intensity is diminished, yet the emission wavelength is red-shifted towards 350 nm in comparison to 330 nm in the folded state (Monsellier and Bedouelle 2005). In addition to the high sensitivity of Trp emission maximum and intensity to its local environment, Trp quantum yield can vary from 0.35 to 0.01 in different protein environments (Eftink 1991). The signal change is further modulated by a shift in the fluorescence emission properties of the seven tyrosine (Tyr) residues per GlpF monomer (Zhdanova et al. 2017; Ghisaidoobe and Chung 2014). As explained in the legend of Fig. 1, Trp and Tyr residues are located at different interfaces, allowing to detect both tertiary and quaternary structural changes. While Tyr possesses a quantum yield that is nearly equivalent to that of Trp, in native proteins, Tyr emission is often quenched, presumably by its interaction with the peptide chain or via energy transfer to Trp (Chen and Barkley 1998). As protein unfolding may result in partial removal of Tyr quenching and Tyr residues are more uniformly distributed in protein structure, Tyr fluorescence is a prospective indicator of overall protein conformational changes (Zhdanova et al. 2016; Cowgill 1968). Additionally, Tyr has been observed to be more sensitive to the specific binding of surfactant molecules compared to Trp residues (Zhdanova et al. 2015).

**Fig. 3:**
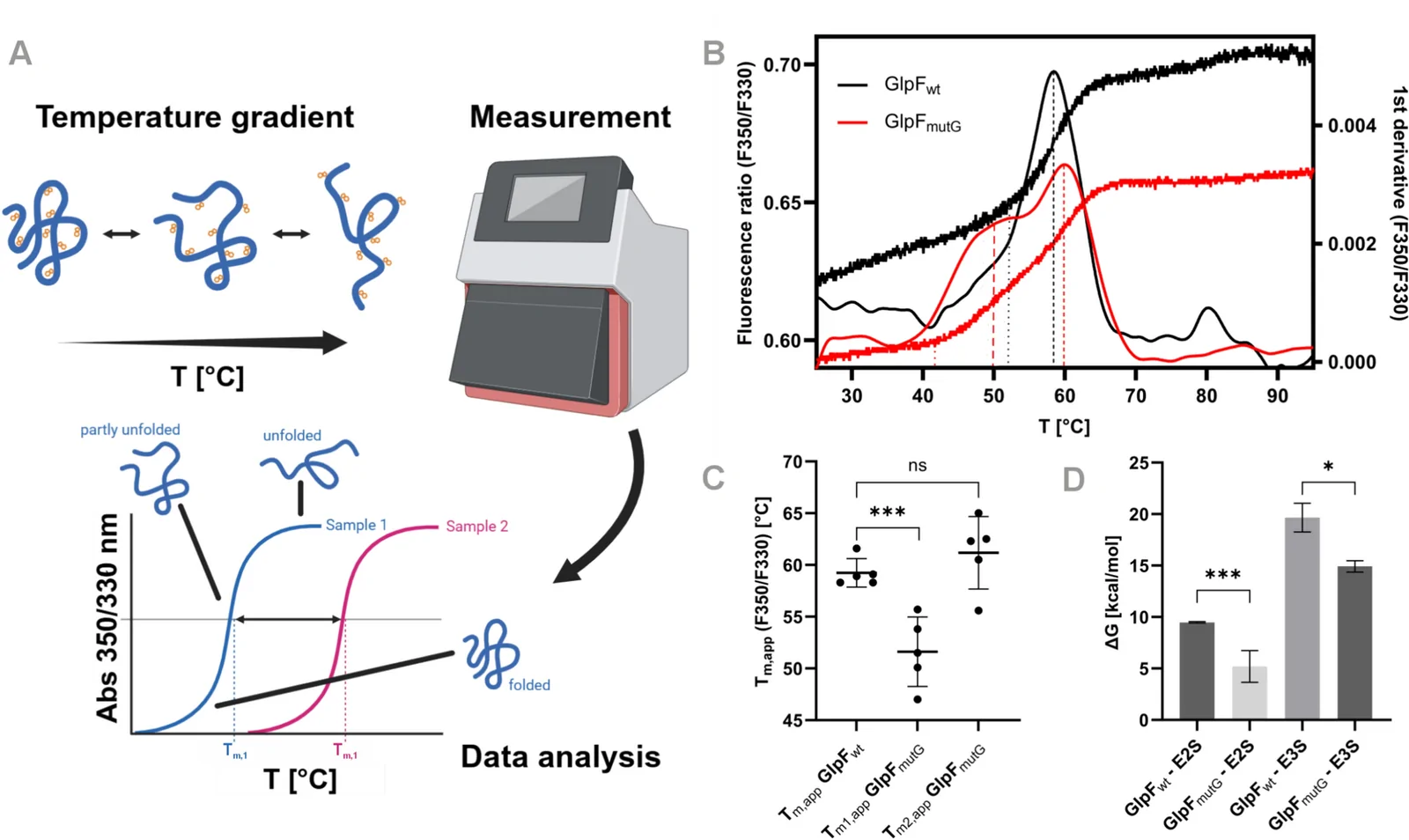
The thermostability of GlpF_wt_ and GlpF_mutG_. **A** The scheme on the left illustrates the working principle of nanoDSF. Protein samples are subjected to a temperature gradient of 1 °C per minute. The thermal unfolding of a protein is quantified by measuring the change in fluorescence intensity of intrinsic tryptophan and tyrosine residues. Melting temperature (T_m_) is defined as the temperature along a thermal denaturation gradient at which 50% of a protein is folded and 50% is unfolded. The temperature at which unfolding commences, designated as T_on_, is an additional parameter indicative of thermal stability. **B** The F_350_/F_330_ ratio of GlpF_wt_ (black line) and GlpF_mutG_ (red line) plotted against the thermal gradient. The peaks in the first derivative correspond to the inflection points in the F_350_/F_330_ ratio plot. The vertical dashed lines indicate the apparent T_m_ values for the two-fold transition of GlpF_mutG_ and the one-fold transition of GlpF_wt_. The vertical dotted lines indicate the T_on_ values. **C** Mean apparent T_m_ values ± SD for data sets including individual values for each independent measurement (n = 5). **D** The apparent Gibbs free energy of unfolding of GlpF_wt_ and GlpF_mutG_, obtained by either the two-state model of unfolding (E2S) or the three-state model (E3S) of unfolding. The mean values are presented with their corresponding standard deviations. The complete data sets, comprising the fits and the associated fit parameters, are presented in Figs. S4 and S5. *** indicates p < 0.001; * indicates p < 0.05; ns indicates that the result is not statistically significant

The plot of the F_350_/F_330_ ratio for GlpF_wt_ against the temperature of a thermal gradient yielded a graph with a single inflection point at an apparent transition temperature (T_m,app_) of 59.2 ± 1.4 °C (Fig. 3). Interestingly, the same graph yielded two inflection points for GlpF_mutG_, namely T_m1,app_ and T_m2,app_ at 51.6 ± 3.4 °C and 61.2 ± 3.5 °C, respectively. While T_m2,app_ of GlpF_mutG_ resembles T_m,app_ of GlpF_wt_, T_m1,app_ of GlpF_mutG_ is lower by approximately 10 °C. This data demonstrates that the conformational stability of GlpF in OG micelles is lost above 52 °C, whereas the onset of unfolding for GlpF_mutG_ occurs already at 42 °C (Fig. 3). Taken together with the above-observed oligomer destabilization of GlpF_mutG_, we speculate that its observed two-fold transition corresponds first to the loss of the quaternary structure and at T_m1,app_ of the tertiary GlpF_mutG_ structure, while the wt lost both at the same melting temperature. The apparent melting temperatures of the GlpF proteins agree well with the range of previously published values for various AQPs in detergent micelles. For example, the T_m,app_ values were found to be 47.0 °C (*h*AQP0 in OG micelles (Swamy-Mruthinti et al. 2013)), 58.0 °C (*So*PIP2;1 in OG micelles (Plasencia et al. 2011)), 60.2 ± 0.2 °C (*h*AQP10 in dodecyl maltoside (DDM) micelles (Oberg et al. 2011)), and 70.7 ± 0.3 °C or 74,5 ± 1.7 °C (*h*AQP2 in octyl glucose neopentyl glycol micelles (Hagstromer et al. 2023; Roche et al. 2017)).

Moreover, fitting the data by the two-state unfolding model enabled us to ascertain the Gibbs free energy of unfolding. The estimated ΔG_wt_ was found to be 9.5 ± 0.9 kcal/mol, while the ΔG_mutG_ was determined to be 5.4 ± 1.5 kcal/mol. Therefore, the change in ΔG between the two protein variants, ΔΔG, indicates that approximately 5 kcal/mol less free energy is required to unfold GlpF_mutG_ in comparison to the wild-type protein (Fig. 3). Additionally, we have calculated the ΔG values using the three-state unfolding model. Despite a relative increase in the ΔG values of approximately 10 kcal/mol, the ΔΔG remained at approximately 5 kcal/mol. For comparison, ΔG of thermal unfolding of soluble proteins lysozyme and RNAse A were measured previously to amount to 14.3 ± 0.2 kcal/mol and 15.2 ± 1.1 kcal/mol, respectively (Kotov et al. 2021).

**Fig. 4:**
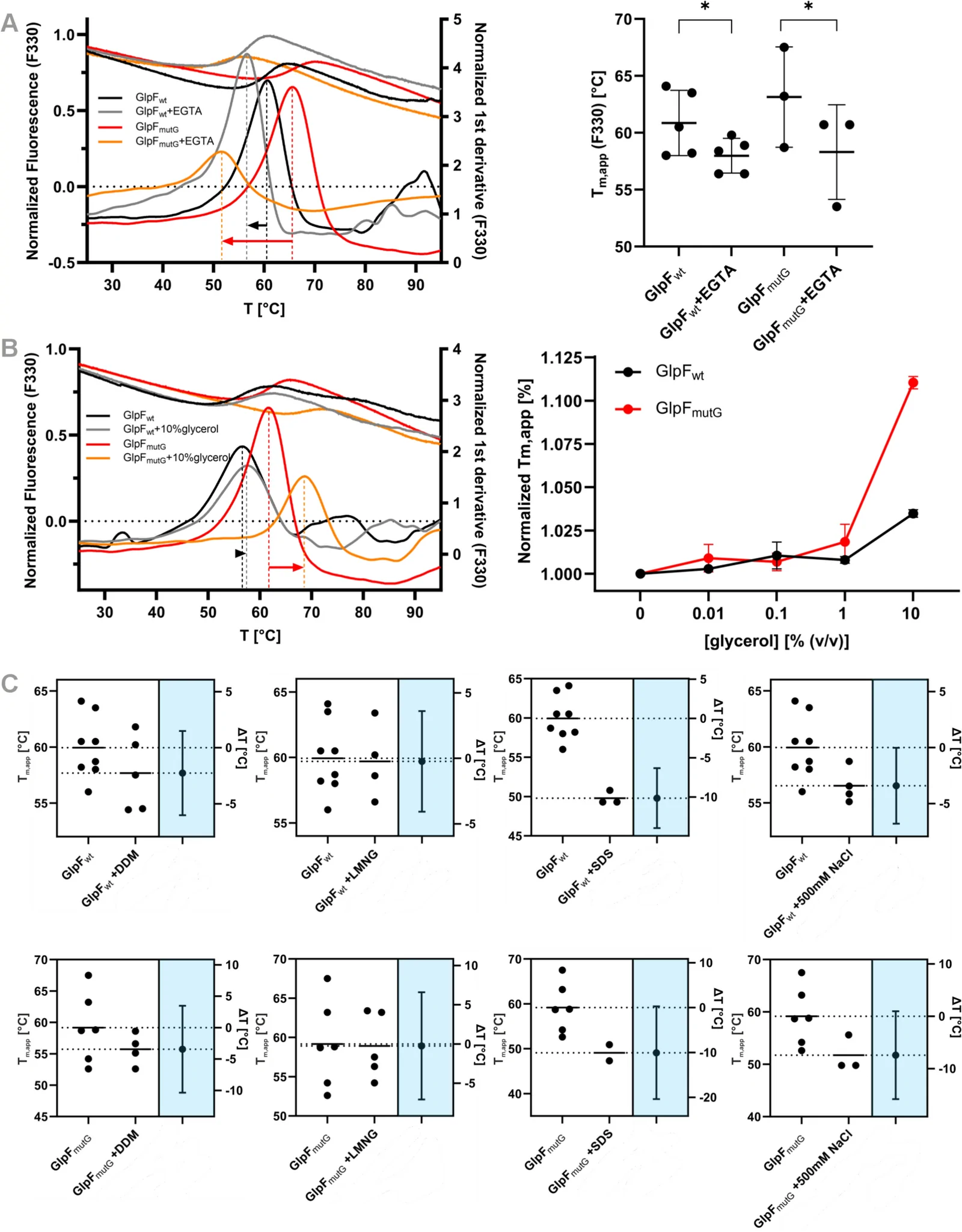
The thermostability of GlpF in the presence of external factors. **A** The impact of divalent ions on the stability of GlpF. The exemplary thermal unfolding (F_330_) and first derivative data of GlpF_wt_ and GlpF_mutG_ are presented in the presence of EGTA with grey and orange lines, respectively, and with black and red lines in the absence of EGTA. The apparent unfolding temperature values are presented as mean + standard deviation values (n = 5 for the wt, n = 3 for mutG). **B** The impact of glycerol on the structural stability of GlpF. As in A, the exemplary thermal unfolding (F_330_) and first derivative data of GlpF_wt_ and GlpF_mutG_ are presented in the presence of 10% (v/v) glycerol (grey and orange lines) and in the absence of glycerol (black and red lines), respectively. Moreover, the normalized relative apparent transition temperature values are plotted against the glycerol concentration as mean + standard deviation (n = 3 for each concentration and variant). **C** Thermal unfolding data of GlpF_wt_ (top row) and GlpF_mutG_ (bottom row) in the presence of 2% DDM, 2% LMNG, 2% SDS, and 500 mM NaCl. Mean apparent melting temperature with standard deviation over n replicas and the temperature shift (ΔT) values are provided

The crystal structure of GlpF (PDB-ID: 1FX8) reveals the presence of a Mg^2+^ ion within the central fifth pore of GlpF, situated in close proximity to the ring of four tryptophan (W42) and four glutamic acid (E43) residues. These residues have been linked in the past to the oligomerization propensity, function, and stability of GlpF (Cymer et al. 2010). To investigate the potential influence of divalent ions on GlpF stability and oligomerization, the thermal unfolding of GlpF_wt_ in OG micelles was monitored in the presence and absence of EGTA, a divalent ion chelator with high affinity for Ca^2+^ and Mg^2+^ ions (Fig. 4A). The depletion of divalent ions from the GlpF tetramers after purification resulted in reduced melting temperatures of both GlpF variants. In detail, the melting temperature decreased by 3 °C and 5 °C for GlpF_wt_ and GlpF_mutG_, respectively (see Fig. S6).

Because the addition of external additives may have strong influence on the F_350_ data (Zhdanova et al. 2015) as explicated above and thus may disable reliable fitting of F_350_/F_330_, we have tested the possibility to obtain the melting temperature purely from F_350_ data. The comparison of the F_350_/F_330_ transitions with the F_330_ transition of GlpF_wt_ and GlpF_mutG_ (shown in Fig. S7) shows that the two-fold transition for GlpF_mutG_ is only discernible in the F_350_/F_330_ ratio. However, it is still possible to ascertain general stabilizing or destabilizing effects on protein structure from the F_330_ data. Therefore, we use the F_330_ raw data to analyze the general effects on both GlpF_wt_ and GlpF_mutG_, for the remainder of the study.

**Fig. 5:**
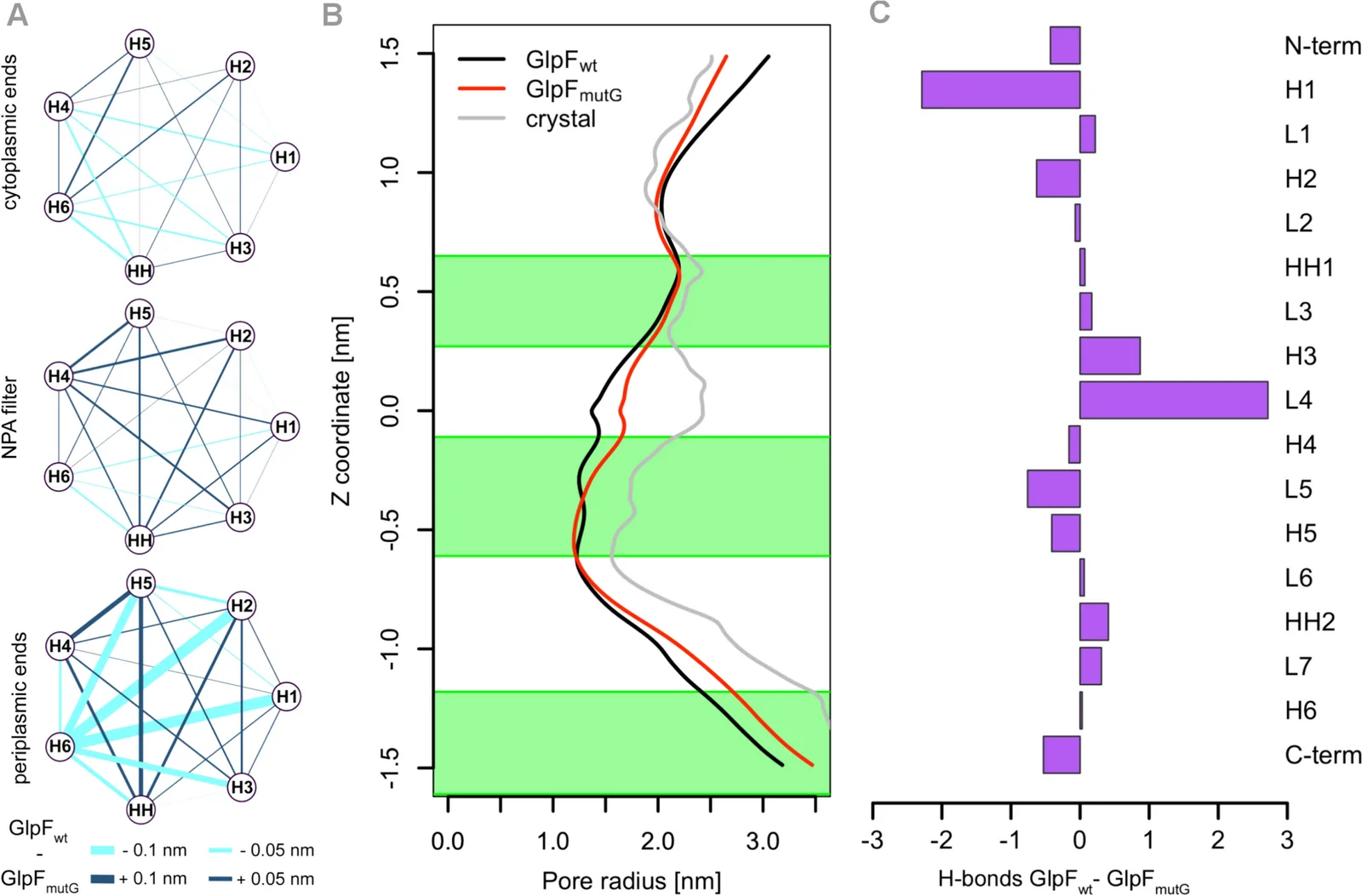
Structural differences between GlpF_wt_ and GlpF_mutG_. **A** Differences in the distances among transmembrane helices at the cytoplasmic side (top), NPA region at the protein center (middle), and at the helix ends at the periplasmic side of the proteins (bottom) between GlpF_mutG_ and GlpF_wt_. Helix distance increase in GlpF_mutG_ relative to GlpF_wt_ is colored dark blue, whereas a distance decrease is colored in cyan. **B** Pore radii along the membrane normal of the GlpF crystal structure (PDB-ID: 1FX8) and the membrane-embedded GlpF_wt_ and GlpF_mutG_ (averaged over 2 simulations, 4 chains each, 1–3 µs simulation time for both variants). Green areas indicate the positions of the three glycerol molecules in the crystal structure. Z-coordinates − 1, 0, and 1 correspond to the respective layers in A. **C** H-bond variation between GlpF_mutG_ and GlpF_wt_ was assessed for the individual secondary structure elements of GlpF

The aqua(glycerol)porin GlpF is responsible for the passive permeability of small neutral substances, including glycerol and water, across the plasma membrane of *E. coli*. In accordance with its biological function, the crystal structure of GlpF revealed the presence of three glycerol molecules at 15% (v/v) glycerol in the crystallization buffer (Fu et al. 2000). One is engaged in two hydrogen bonds with the highly conserved arginine at position 206, which constitutes part of the aromatic and Arg-containing Ar/R selectivity filter. The second glycerol molecule is situated at the NPA filter, where it is stabilized by two hydrogen bonds with the asparagines at positions 68 and 203 of GlpF. The third molecule is located at the periplasmic ends of the transmembrane helices. To ascertain the impact of the natural substrate glycerol on the thermal stability of GlpF_wt_ and GlpF_mutG_, the T_m,app_ values were determined at varying glycerol concentrations, ranging from 0% (v/v) to 10% (v/v) in four logarithmically spaced steps (see Fig. S8). Figure 4B illustrates the stabilization of GlpF_wt_ and GlpF_mutG_ with increasing glycerol concentration. The T_m,app_ of GlpF_wt_ slightly increases by 3.5% from 58.4 ± 1.3 °C to 60.4 ± 1.4 °C, whereas the increase for GlpF_mutG_ is three times as high, i.e. by 11%. Namely, the T_m,app_ of GlpF_mutG_ rises from 55.2 ± 1.9 °C in the absence of glycerol to 61.3 ± 2.1 °C with 10% (v/v) glycerol, with the majority of the stabilization occurring above 1% (v/v). A possible explanation for this effect is the collapse of the GlpF pore in the absence of glycerol as seen in MD simulations and depicted in Fig. 5B.

Finally, we assessed the effect of detergents SDS, DDM, and lauryl maltose neopentyl glycol (LMNG), as well as the role of NaCl concentration on the thermal stability of GlpF. To this end, 2% of the respective detergent was titrated to the GlpF_wt_ or GlpF_mutG_-containing OG micelles. Furthermore, the respective T_m,app_ values were estimated at physiological (100 mM) and high salt (500 mM) NaCl concentrations. As illustrated in Fig. 4C, there is no significant change in the proteins' stability compared to pure OG when 2% DDM or 2% LMNG are used. In contrast, the addition of 2% SDS or unphysiologically high concentrations of NaCl resulted in a significant reduction in T_m,app_, by 10 °C for both variants and 3.5 °C and 7.5 °C for GlpF_wt_ and GlpF_mutG_, respectively.

In order to comprehend the molecular underpinnings that underlie the destabilization of GlpF_mutG_ compared to the GlpF_wt_, all-atom molecular dynamics simulations of both tetrameric variants in PLE lipids were employed (Goessweiner-Mohr et al. 2022; Pluhackova and Horner 2021; Pluhackova et al. 2022). Notably, no Mg^2+^ ion and glycerol were present in the simulations. The analysis reveals that all four studied Cys residues form H-bonds (Carugo 2023) with multiple residues within the same protomer of GlpF_wt_ (see Table S1). Cys11, Cys99, and Cys80 exhibited a high propensity for H-bonding, forming H-bonds with Leu7, Ala95, and Phe24 in 84.5%, 71.0%, and 11.5% of the analyzed simulation time (combined data over two replicas, 1–3 µs), respectively. Furthermore, Cys28 forms an H-bond with the backbone of Ala192 in the neighboring protomer 27.2% of the analyzed time (see Fig. S9). Mutation of these cysteines into glycines leads to slightly different adaptation of the tertiary GlpF structure as depicted in Fig. 5A. The relative movements of the center of mass of transmembrane helices of GlpF_mutG_ in relation to the GlpF_wt_ at three planes in the membrane (cytoplasmic and periplasmic ends of the helices and the NPA region) illustrate the respective structural rearrangements. The periplasmic vestibule demonstrates the most significant alterations with the periplasmic end of H6 moving towards the pore in the GlpF_mutG_ compared to GlpF_wt_ (Fig. 5A). The NPA region undergoes a slight opening in GlpF_mutG_, while the differences at the cytoplasmic side are marginal. The pore radius (average of 2 replicas for each variant, 1–3 µs simulation time was used for the analysis), as depicted in Fig. 5B, reveals small variations along the pore axis between both variants. At z = 0 (NPA region) the slight opening of the tertiary structure estimated in Fig. 5A middle is reflected in a slightly larger pore radius for GlpF_mutG_ compared to GlpF_wt_. At the same time, major narrowing of the glycerol-free pores of both GlpFs as compared to the glycerol-containing GlpF crystal structure (PDB-ID: 1FX8) is observed.

A thorough analysis of the total number of H-bonds in the secondary structure segments of the two protein variants (see Fig. 5C) substantiates the hypothesis of structural rearrangements due to the cysteine to glycine mutations. Besides substantial variations in the overall number of H-bonds of secondary structural elements between GlpF_wt_ and Glpf_mutG_, a decline of 0.4 and 0.2 H-bonds in the overall number of H-bonds within the tetramers and the intra-protomer H-bonds was observed, respectively. These observations are further substantiated by the interaction energy (comprising both electrostatics and van der Waals contributions) between protomers, which is estimated to be − 763 ± 13 kcal/mol and − 721 ± 4 kcal/mol for GlpF_wt_ and GlpF_mutG_, respectively. At the same time, the intrachain electrostatic energy and van der Waals energy of GlpF_mutG_ become less stabilizing. In detail, whereas the electrostatic energy alters not significantly from −16,637 ± 8 kcal/mol (GlpF_wt_) to − 16,625 ± 12 kcal/mol (GlpF_mutG_), the van der Waals energy changes from − 1666 ± 4 kcal/mol for GlpF_wt_ to − 1650 ± 3 kcal/mol for GlpF_mutG_ (see Fig. S10). To elucidate if these changes are solely based on the removal of the cysteine side chains or indicative of a structural rearrangement, we have exemplarily calculated the intra- and interchain interaction energies of the final structure of a wt simulation in the wt form and upon introduction of the four glycine mutations. The analysis revealed that the wt's van der Waals and electrostatic energies within a single protomer are by − 19 kcal/mol and −8 kcal/mol more stabilizing, respectively, than in the mutant. These values are comparable with the above-described differences in intrachain van der Waals and electrostatic energies between the GlpF_wt_ and GlpF_mutG_ simulations, amounting to − 12 kcal/mol and − 16 kcal/mol, respectively. However, the analysis of the interchain energies in the tetrameric assembly has revealed that the artificial introduction of the four glycines results in a destabilization of only 2 kcal/mol (electrostatics) and 6 kcal/mol (van der Waals energy) which is less than the destabilization of 9 kcal/mol (electrostatics) and 32 kcal/mol (van der Waals energy) in the mutant compared to the wt simulations. This suggests a structural rearrangement of the interface between the individual chains in the GlpF_mutG_ simulations compared to the wt. Notable, a direct comparison of the interaction energies under consideration with the experimentally determined ΔΔG of approximately 5 kcal/mol is not appropriate, as these energies constitute only a part of the enthalpic energy. Additionally, entropic contributions are absent, and, most notably, in silico absolute energies are contingent on the applied force field from simulations. Despite the lack of evidence for the involvement of the four Cys residues in disulfide bond formation (see Fig. S11), a tool often used by nature to stabilize the protein fold (Creighton 1988), our MD simulations reveal that subtle structural changes caused by the substitution of the cysteines by glycines together with the changes in the H-bond network stabilizing GlpF_wt_, destabilize GlpF_mutG_ both intramolecularly and within the tetramer. In order to ascertain whether this finding can be generalized to other hydrogen bond acceptors/donor sites, such as Thr or Ser, further studies on various wild-type and mutant proteins are required.

Glycine residues are frequently viewed as helix breakers. However, our analysis shows that GlpF exhibits 32 naturally occurring glycine residues in its primary sequence (see Fig. S12), 19 of which are located within transmembrane helices. MD simulations reveal no indication of Gly-induced helix unfolding or bending (see Fig. S13 and Table S2). Hence, the additional 4 glycine residues are not acting as helix breaker in GlpF.

## Conclusion

The present study underscores the capacity of glycine substitutions to disrupt the structural integrity of GlpF, corroborating the findings of mutational investigations conducted on hen egg white lysozyme (Masumoto et al. 2000). Nevertheless, the majority of studies have indicated that residue mutations to glycine result in enhanced protein stability (Stites et al. 1994; Trevino et al. 2007; Lu et al. 2022; Stojanoski et al. 2016). Furthermore, our data demonstrate that the impact of external modifiers, such as divalent ions and the substrate glycerol, on the stability of GlpF is contingent upon the channel's overall stability. The stabilizing effect was more pronounced for the variant with reduced stability. Similarly, lipids were demonstrated to exhibit an increased stabilizing effect on loosely interacting protomers (Gupta et al. 2017). In conjunction with the study conducted by Kozono et al. (2003) (Kozono et al. 2003), our data illustrate the importance of the lipid bilayer for the stability of membrane proteins. The structural unfolding temperature of purified GlpF in detergent micelles is lowered by approximately 20 °C compared with T_m,app_ of approximately 80 °C for functional GlpF reconstituted in a lipid membrane (Kozono et al. 2003). This demonstrates the significant impact of specific lipid interactions and the global physicochemical properties of the lipid bilayer on the structure and stability of membrane proteins.

## Supplementary Information

Below is the link to the electronic supplementary material.

Supplementary file1 (DOCX 4364 kb)

## Data Availability

Data used to perform MD simulations are deposited in the DaRus repository https://doi.org/10.18419/DARUS-5324. Experimental data substantiating the findings of this study are deposited in the Zenodo repository https://doi.org/10.5281/zenodo.17423375.
