# Supplementary material for: Cysteine-mediated structural stabilization of the tetrameric GlpF
Source: Eur Biophys J. 2025 Nov 3;55(5):847–60. doi: 10.1007/s00249-025-01803-6 (PMC13661670; doi:10.1007/s00249-025-01803-6)
Supplement: Supplementary file 1 [file 249_2025_1803_MOESM1_ESM.docx]

**Supplementary Information**

**Cysteine-mediated structural stabilization of the tetrameric GlpF**

Christine Siligan^1*^, Sascha Gratzl^1*^, Kristyna Pluhackova^2^, Nikolaus Goessweiner-Mohr^1^, Peter Pohl^1^, Andreas Horner^1#^

^1^ Institute of Biophysics, Johannes Kepler University Linz, Austria; ^2^ Stuttgart Center for Simulation Science, Cluster of Excellence EXC 2075, University of Stuttgart, Germany;
^*^ These authors contributed equally to the work.
^#^ Corresponding author: andreas.horner@jku.at

***Figure S1. A comparative structural analysis of the GlpF crystal structure and the AlphaFold3 model.*** *The tetrameric assembly from the lateral view, with the periplasmic side oriented upwards and the cytoplasmic side facing downwards is shown on the left. The top view of GlpF is presented from the perspective of the periplasmic side on the right. The AlphaFold3 model in green reveals additional structural features at the N- and C-termini not seen in the crystal structure in blue (PDB-ID: 1FX8).*

***Figure S2. Western blot of GlpF oligomer structures in E. coli solubilized membrane protein samples.*** *His-tagged GlpF was overexpressed in E. coli cells and membrane proteins were solubilized with OG. Samples were split and one part was treated with urea, DTT, and NEM (denatured sample; D) before separation by SDS-PAGE and further analysis by Western blotting using αHis antibodies. While GlpF_wt_ is present in higher oligomeric states under untreated (semi-native; N) and denatured (D) conditions, GlpF_mutG_ is only present in its monomeric form.*

***Figure S3. Chemostability of GlpF_wt_ and GlpF_mutG_.*** *Purified and AF647-labeled GlpF_wt_ and GlpF_mutG_ were incubated in the presence of 2-4 % OG or 0.5-2 % SDS for 15 minutes before cross-linking the oligomers with 0.0625 % glutaraldehyde for 5 minutes. Samples were separated by SDS-PAGE and visualized. While GlpF_wt_ oligomers are stable even in the presence of high concentrations of detergents, GlpF_mutG_ is destabilized with increasing amounts of detergent.*

| $k_{n}$  [1] | | | $b_{n}$  [°C] | | | $k_{n}$  [1] | | | $b_{u}$  [°C] | | | $\Delta H_{m}$ [kcal/mol] | | | $T_{m}$  [°C] | | |
| --- | --- | --- | --- | --- | --- | --- | --- | --- | --- | --- | --- | --- | --- | --- | --- | --- | --- |
| 1,3E-03 | ± | 4,9E-05 | 0,23 | ± | 0,006 | 1,7E-04 | ± | 1,0E-05 | 0,63 | ± | 0,007 | 87,3 | ± | 2,5 | 58,3 | ± | 0,01 |
| 1,0E-03 | ± | 3,3E-05 | 0,32 | ± | 0,010 | 4,7E-04 | ± | 1,8E-05 | 0,53 | ± | 0,006 | 104,0 | ± | 3,3 | 58,3 | ± | 0,02 |
| 8,3E-04 | ± | 2,2E-05 | 0,38 | ± | 0,017 | 3,3E-04 | ± | 2,9E-05 | 0,59 | ± | 0,005 | 97,4 | ± | 2,7 | 58,9 | ± | 0,02 |
| 8,2E-04 | ± | 5,3E-06 | 0,40 | ± | 0,001 | 4,7E-04 | ± | 1,3E-05 | 0,53 | ± | 0,005 | 97,3 | ± | 1,1 | 61,0 | ± | 0,01 |
| 7,3E-04 | ± | 4,0E-06 | 0,41 | ± | 0,001 | 4,8E-04 | ± | 1,7E-05 | 0,53 | ± | 0,006 | 81,3 | ± | 2,0 | 59,1 | ± | 0,01 |

***Figure S4. Thermal unfolding data (F_330_/F_350_) for GlpF_wt_, fitted with the two-state equilibrium model.*** *The fit parameters corresponding to the fit curves (shown in top 2 rows) in red are summarized in the Table at the bottom.*

| $k_{n}$  [1] | $b_{n}$  [°C] | $k_{u}$  [1] | $b_{u}$  [°C] | $k_{I}$  [1] | $\Delta H_{m1}$  [kcal/mol] | $T_{1}$  [°C] | $\Delta H_{m2}$  [kcal/mol] | $T_{2}$  [°C] |
| --- | --- | --- | --- | --- | --- | --- | --- | --- |
| 8,82E-05 | 0,60 | 5,71E-04 | 0,50 | 7,34E-01 | 35,1 | 62,0 | 102,0 | 62,2 |
| 9,70E-04 | 0,32 | 2,29E-04 | 0,61 | 6,91E-01 | 60,3 | 58,7 | 101,0 | 62,0 |
| 7,07E-04 | 0,42 | 5,04E-03 | 0,53 | 7,06E-01 | 73,0 | 60,2 | 55,9 | 65,2 |
| 6,21E-05 | 0,62 | 3,92E-04 | 0,56 | 6,70E-01 | 21,0 | 39,0 | 78,2 | 59,8 |
| 5,53E-05 | 0,60 | 5,11E-04 | 0,52 | 6,80E-01 | >100 | 55,0 | 81,8 | 57,6 |
|  |  |  |  |  |  |  |  |  |
| Error [%] | | | | | | | | |
| $k_{n}$ | $b_{n}$ | $k_{u}$ | $b_{u}$ | $k_{I}$ | $\Delta H_{m1}$ | $T_{1}$ | $\Delta H_{m2}$ | $T_{2}$ |
| >100 | 11,0 | 7,1 | 2,9 | 3,40 | 21 | 0,76 | 22,0 | 0,41 |
| 6,2 | 5,7 | 9,5 | 1,3 | 0,83 | 13 | 0,29 | 39,0 | 0,35 |
| 6,0 | 3,1 | 17,0 | 5,9 | 1,30 | 16 | 0,46 | 51,0 | 1,60 |
| >100 | 27,0 | 5,8 | 1,4 | 0,46 | 21 | 2,20 | 4,1 | 0,07 |
| >100 | 67,0 | 3,5 | 1,2 | 8,40 | >100 | 6,70 | 9,7 | 0,36 |

***Figure S5. Thermal unfolding data (similar to Figure S4) for GlpF_wt_, fitted with the three-state equilibrium model.*** *The fit parameters corresponding to the fit curves (shown in top 2 rows) in red are summarized in the Table at the bottom.*

***Figure S6. The impact of divalent cations on the structural stability of GlpF.*** *Mean apparent T_m_ values ± SD for data sets including individual values for each independent measurement (n = 5 for GlpF_wt_, n = 3 for GlpF_mutG_). The mean and standard deviation of the destabilization temperatures (ΔT) resulting from divalent ion depletion via the addition of 1mM EGTA for GlpF_wt_ and GlpF_mutG_ are depicted on the right side of the graphs.*

***Figure S7. A comparative analysis of the first derivative of the F_350_/F_330_ ratio and of the F_330_ value for GlpF_wt_ and GlpF_mutG_.*** *The double peak observed for GlpF_mutG_ is evident only in the first derivative of the F_350_/F_330_ ratio.*

***Figure S8. The impact of glycerol on the structural stability of GlpF.*** *Top: Mean and standard deviation (n = 3) for the apparent transition temperature (T_m,app_) are plotted against the glycerol concentration for GlpF_wt_ (black) and GlpF_mutG_ (red). Bottom: Mean apparent T_m_ values ± SD for data sets including individual values for each independent measurement (n=3). For ΔT also the mean and standard are provided. ** indicates p < 0.001*

|  | Acceptor | |  |  |
| --- | --- | --- | --- | --- |
|  | Residue | Atom | Probability [%] | stdev  [%] |
| Cys11 HG | Pro88 | O | 7,24 | 2,49 |
|  | Ser92 | OG | 9,96 | 2,96 |
|  | Leu7 | O | 84,85 | 2,88 |
|  | Cys11 | N | 60,42 | 2,16 |
| Cys28 HG | Phe24 | O | 11,46 | 7,59 |
|  | Gly25 | O | 9,71 | 7,03 |
|  | Cys28 | N | 7,45 | 4,80 |
|  | Cys28 | O | 31,52 | 5,45 |
|  | Val29 | N | 1,21 | 0,67 |
| Cys80 HG | Leu77 | O | 5,08 | 4,02 |
|  | Phe78 | O | 6,86 | 5,40 |
|  | Ala79 | O | 3,75 | 0,95 |
|  | Cys80 | N | 26,21 | 3,45 |
|  | Cys80 | O | 6,26 | 1,49 |
|  | Asp262 | O | 1,05 | 3,10 |
|  | Val265 | O | 1,72 | 4,57 |
| Cys99 HG | Ala95 | O | 71,06 | 9,92 |
|  | Cys99 | N | 48,52 | 7,00 |
|  | Cys99 | O | 11,08 | 4,26 |

***Table S1. Intrachain H-bonds of Cys residues in GlpF_wt_.*** *Only H-bond acceptors exhibiting an interaction probability over the analyzed simulation time (2 replicas, 4 chains each, 1-3 µs) of more than 1 % are shown. The H-bonding probability is defined as the fraction of the simulation time in which the S-H hydrogen is closer than 0.3 nm to the acceptor. The standard deviation is estimated per chain per µs for the two simulation replicas analyzed over 1-3µs (n=16).*

***Figure S9. Interchain H-bonds of Cys28 in GlpF_wt_.*** *Cys28 forms in 27 % of the simulation time an H-bond with Ala192 of the neighboring protomer. In less than 0,5 % of the time, Cys28 forms an H-bond with Ser193. The error bars denote SEM estimated over 1 µs intervals per chain (n=16).*

***Figure S10. Intrachain interaction energies.*** *Both intrachain electrostatic energy and van der Waals energy are less favorable in GlpF_mutG_ as compared to GlpF_wt_. Values are given as averages ± SEM estimated over individual chains and 1 µs intervals over 2 replica simulations, 4 chains each and simulation time 1-3 µs (n=16). A two-tailed test was conducted to analyze the intrachain electrostatics and van der Waals energy of GlpF_mutG_ versus GlpF_wt_. The results of these tests were 0.42 and 0.0019, respectively.*

***Figure S11. Effect of reducing β-mercaptoethanol and high temperature on GlpF stability.*** *GlpF constructs were overexpressed in E. coli cells and purified in the absence and presence of β-mercaptoethanol. After labeling with AF647, samples purified in the absence of β-mercaptoethanol were incubated in β-mercaptoethanol for 15 minutes at RT or 95 °C. All samples were subjected to SDS-PAGE and visualized. No difference can be seen between untreated samples and samples purified in the presence of or incubated with β-mercaptoethanol, while aggregation can be seen when samples are exposed to 95 °C.*

***Figure S12. Distribution of naturally occurring glycine residues in wild-type GlpF.*** *19 out of 32 glycine residues (colored orange) are located within transmembrane helices.*

***Figure S13.*** ***MD simulations reveal no impact of Gly residues on the secondary structure of GlpF_mutG_.*** *Whereas C11G is located at the beginning of H1, C28/99G residues are located in the middle of H1 and H3, respectively, and C80G is part of the cytoplasmic loop connecting HH1 and H3. C11/28/80/90G residues are shown as red spheres. GlpF_mutG_ simulation snapshots (extracted every 100 ns) are aligned in PyMOL and shown as colored cartoon.*

|  | *GlpF_wt_* | *GlpF_mutG_* |
| --- | --- | --- |
| *r11* | *1.94 ± 0.01* | *1.38 ± 0.11* |
| *r28* | *1.84 ± 0.01* | *1.77 ± 0.01* |
| *r80* | *0.15 ± 0.02* | *0.23 ± 0.05* |
| *r99* | *1.93 ± 0.01* | *1.91 ± 0.01* |

***Table S2. Effect of glycine residues on secondary structural elements.*** *Average number of hydrogen bonds the protein backbone of residues 11, 28, 80, and 99 forms with the backbone of the other residues in the same chain (2 replicas, 4 chains each, 1-3 µs). Error is given as SEM over individual chains. Whereas residue position 11 is located at the beginning of H1, r28 and r99 reside in the middle of H1 and H3, respectively, and r80 is part of the cytoplasmic loop connecting HH1 and H3. For comparison, two H-bonds per backbone of one residue are formed in a central part of a perfect* *α-helix.*
